# Cerebroprotective Effect against Cerebral Ischemia of the Combined Extract of *Oryza sativa* and *Anethum graveolens* in Metabolic Syndrome Rats

**DOI:** 10.1155/2019/9658267

**Published:** 2019-11-11

**Authors:** Jintanaporn Wattanathorn, Warin Ohnon, Wipawee Thukhammee, Supaporn Muchmapura, Panakaporn Wannanon, Terdthai Tong-un

**Affiliations:** ^1^Department of Physiology, Faculty of Medicine, Khon Kaen University, Khon Kaen 40002, Thailand; ^2^Integrative Complementary Alternative Medicine Research and Development Center, Khon Kaen University, Khon Kaen 40002, Thailand; ^3^Research Institute for Human High Performance and Health Promotion, Khon Kaen University, Khon Kaen 40002, Thailand; ^4^Department of Physiology and Graduate School (Neuroscience Program), Faculty of Medicine, Khon Kaen University, Khon Kaen 40002, Thailand

## Abstract

The novel strategy against ischemic stroke in metabolic syndrome (MetS) targeting at oxidative stress and inflammation has gained attention due to the limitation of the current therapy. Due to the antioxidant and anti-inflammation of the combined extract of *Oryza sativa* and *Anethum graveolens*, the cerebroprotective effect against cerebral ischemia in MetS condition has been focused. Since no data were available, this study was set up to determine the effects of the combined extract of *Oryza sativa* L. and *Anethum graveolens* Linn. against ischemic stroke in the animal model of metabolic syndrome. The possible underlying mechanism was also further investigated. Male Wistar rats (180-220 g) were fed with high-carbohydrate high-fat diet (HCHF diet) to induce metabolic syndrome-like condition. Then, MetS rats were subjected to reperfusion injury at the right middle cerebral artery. The combined extract of *O. sativa* and *A. graveolens* (OA extract) at doses of 0.5, 5, and 50 mg/kg BW was fed once daily for 21 days. Neurological assessment was performed every 7 days throughout the experimental period. At the end of study, brain infarction volume, neuron and glial fibrillary acidic protein- (GFAP-) positive cell density, the oxidative stress status, the expressions of proinflammatory cytokines (NF-*κ*B, IL-6), and eNOS in the cortical area together with the expression of VCAM-1 and the histological changes of common carotid artery were determined. It was found that OA extract decreased brain infarction, neurological score, oxidative stress status, and inflammatory mediators but increased eNOS expression in the cortical area; the increased VCAM-1 and intima-media thickness together with the reduction of lumen diameter of common carotid artery of MetS eats with MCAO were also mitigated by OA extract. These data suggest the cerebroprotective effect of OA, and the underlying mechanism may occur partly via the improvement of oxidative stress status, inflammation, and brain blood supply.

## 1. Introduction

Nowadays, the prevalence of metabolic syndrome, an important noncommunicating disease, is continually rising and it is regarded as the critical health problem in both the developed and developing countries. The prevalence of metabolic syndrome (MetS), a cluster of conditions associated with glucose intolerance, insulin resistance (IR), central obesity, dyslipidemia, and hypertension [1–3], in Asia has increased rapidly and continually. MetS also increases the risk of many deleterious disorders, especially stroke [4].

Stroke has been recognized as a leading cause of morbidity and mortality worldwide. It has been predicted that the number of global stroke deaths should be 7.8 million within 2030 if the effective vascular disease prevention program is not implemented. The most common type of stroke worldwide is ischemic stroke [5]. It has been reported that the pathophysiology of ischemic stroke especially in the aged population is attributed to MetS and atherosclerosis. Since MetS is an important cause of atherosclerosis, a key pathophysiological factor of ischemic stroke [5], the prevalence of ischemic stroke in MetS also increases. Stroke in MetS often induces poor outcomes [6] and produces both financial and psychological burdens for the country. Despite increasing their importance, the effective pharmacological agents which can effectively prevent and treat cerebral ischemia with MetS are still in an unsatisfaction level. Therefore, the novel neuroprotectant is required.

In recent years, many reviews have been published about the effects and potential benefits of herbal medicine in MetS and stroke [7–8]. It has been revealed that some herbal medicines may improve the brain microcirculation, protect against ischemic reperfusion injury, possess neuroprotective properties, and inhibit apoptosis in MetS and ischemic stroke patients [7, 9]. In Thailand, numerous plants are reputed for disease treatment and health promotion. Both *Oryza sativa* L. and *Anethum graveolens* Linn. are also reputed for both actions. In addition, they also possess a potent antioxidant activity [10, 11]. Based on this reputation and the crucial role of oxidative stress on the pathophysiology of MetS and stroke, the possibility of these medicinal plants to attenuate the diseases such as ischemic stroke in MetS condition has gained attention. Since no data concerning this issue are available, this study was set up to determine the effects of the combined extract of *Oryza sativa* L. and *Anethum graveolens* Linn. against ischemic stroke in the animal model of metabolic syndrome. In addition, the possible underlying mechanism was also investigated.

## 2. Materials and Methods

### 2.1. Plant Material Preparation and Extraction

Grains of black sticky rice (*Oryza sativa* L. indica) and aerial part of dill (*Anethum graveolens* Linn.) used in this study were collected from Khon Kaen province in September to October. The voucher specimens (ICAM 12001 and ICAM 12002) were authenticated by Associate Professor Panee Sirisa-ard, the pharmacognosy expert from the Faculty of Pharmacy, Chiangmai University, and kept at the Integrative Complementary Alternative Medicine Research and Development Center, Khon Kaen University. All samples were cleaned and dried in an oven (Memmert GmbH, USA) at 60°C for 72 hours. Following this process, the aqueous extract of *O. sativa* and 95% hydroalcoholic extract of *A. graveolens* were prepared by using the maceration technique for 24 hours at room temperature. The extracts were subjected to a 3000 rpm centrifugation for 10 minutes. Then, they were filtered with a filter paper (Whatman No. 1), dried by using a freeze dryer, and kept at -20°C until used. The yields of *O. sativa* and *A. graveolens* were 10 and 26 percent, respectively. To prepare the combined extract (OA extract), the extracts of *O. sativa* and *A. graveolens* were mixed at a ratio which produced the highest benefit for ischemic stroke in metabolic syndrome according to our pilot study [12].

### 2.2. Determination of Total Phenolic Compound Contents

The Folin-Ciocalteu colorimetric method was used to determine the total phenolic compound content of OA extract [13]. Briefly, 158 *μ*L of distilled water, 20 *μ*L of the tested substances, and 1000 *μ*L of 50% Folin-Ciocalteu phenol reagent (Sigma-Aldrich, USA) were mixed and subjected to an 8-minute incubation period at 37°C. Following this step, an aliquot 20% Na_2_CO_3_ (Sigma-Aldrich, USA) at the volume of 30 *μ*L was added and incubated in the dark room for 2 hours at room temperature. At the end of incubation period, an absorbance at 765 nm was measured. The content of total phenolic compounds was expressed as mg gallic acid equivalent (GAE)/mg sample. Various concentrations of gallic acid (Sigma-Aldrich, USA) ranging from 1 to 500 *μ*g/mL were prepared for a standard reference.

### 2.3. Determination of Total Flavonoid Content

The total flavonoid content of OA extract was determined via the modified aluminium chloride colorimetric method [14]. Briefly, 100 *μ*L of 2% AlCl_3_·6H_2_O (2 g dissolved in 100 mL of methanol) and 100 *μ*L of the tested substances were mixed and incubated for 30 minutes at room temperature. After the incubation period, the absorbance at 415 nm was measured. Results were expressed as *μ*g quercetin equivalent (QE)/mg sample.

### 2.4. Determination of Antioxidant Activities

#### 2.4.1. 1,1-Diphenyl-2-Picrylhydrazyl (DPPH) Assay

This assay was performed based on an ability to scavenge the stable 1,1-diphenyl-2-picrylhydrazyl (DPPH) radical of the tested substances [15]. Briefly, 50 *μ*L of various concentrations ranging from 1, 5, 10, 50, 100, 250, 500, and 1000 *μ*g/mL of OA extract was mixed with 0.25 mL of 0.15 mM DPPH solution. Following this step, the incubation of the mixture for 30 minutes in a dark room was carried out. Then, an absorbance at 517 nm was measured using a Spectronic™ GENESYS™ 20 spectrophotometer (Thermo Electron Corporation, IL, USA). L-Ascorbic acid was used as a standard reference. DPPH^·^ blank solution was prepared by mixing 300 *μ*L DPPH^·^ solution with 2.7 mL of methanol. The percent inhibition of DPPH radical was calculated according to the following formula: %DPPH inhibition = ((*A*_DPPH_‐*A*_extract_)/*A*_DPPH_) × 100. *A*_DPPH_ was the absorbance value of the DPPH· blank solution. *A*_extract_ was the absorbance value of the sample solution. 50% inhibition (IC50) value was calculated using the graph showing the relation between the percent inhibition and the extract concentration [15, 16].

#### 2.4.2. Ferric-Reducing Antioxidant Power (FRAP) Assay

The assessment of FRAP assay was performed based on the ability to change ferric tripyridyltriazine (Fe^3+^-TPTZ) complex to ferrous tripyridyltriazine (Fe^2+^-TPTZ) [17]. In brief, FRAP reagent was freshly prepared by mixing 5 mL of 10 mM TPTZ (Sigma-Aldrich, USA) solution, 5 mL of 20 mM ferric chloride solution (FeCl_3_) (Sigma-Aldrich, USA), and 50 mL of 300 mM acetate buffer, pH 3.6 (Sigma-Aldrich, USA) together. Then, the mixture was mixed with 190 *μ*L of FRAP reagent and 10 *μ*L of samples and incubated for 10 minutes at 37°C. The absorbance was measured at 593 nm, and L-ascorbic acid was used as a standard reference. Results were expressed as the EC50 value.

### 2.5. Determination of Cyclooxygenase 2 (COX-2) Inhibition Activity

The effect of OA extract on the activity of cyclooxygenase 2 (COX-2), an enzyme playing a key role in inflammation, was also determined. Briefly, the mixture containing 150 *μ*L of 100 mM Tris-HCl buffer pH 8.0, 10 *μ*L of 50 nM of COX-2, 10 *μ*L of 0.5 *μ*M of heme, and 10 *μ*L of various concentrations of OA extract was added to a 96-well plate. Then, 20 *μ*L of 10 *μ*M of N,N,N′,N′-tetramethyl-p-phenylenediamine (TMPD) and 20 *μ*L of 100 *μ*M arachidonic acid (Cayman Chemical, USA) were added and incubated for 5 minutes at room temperature. At the end of incubation period, an absorbance at 590 nm was measured using a microplate reader. Indomethacin was used as a standard reference. The percent inhibition of COX-2 was calculated, and the results were expressed as EC50 [18].

### 2.6. Experimental Protocol

Adult male Wistar rats, age 10-14 weeks, weighing 180-220 grams were obtained from the National Laboratory Animal Center, Salaya, Nakhon Pathom, Thailand. Rats were housed in standard metal cages and maintained in 12 : 12-hour light : dark cycle at 22 ± 2°C. Animals were given food and water ad libitum. This study was approved by the Institutional Animal Care and Use Committee, Khon Kaen University, Khon Kaen, Thailand (AEKKU 30/2558).

The experimental animals were divided into 7 groups (*n* = 6/group) as the following. Group I naïve intact: rats were fed with normal diet comprised of 42% carbohydrate, 24% protein, and 4.5% fat and received no treatment. Group II HCHF+sham operation+vehicle: all rats were fed with high-carbohydrate high-fat (HCHF) diet and subjected to sham operation and vehicle treatment. Group III HCHF+MCAO+vehicle: all rats were fed with HCHF diet and subjected to the temporary occlusion of right middle cerebral artery (Rt. MCAO) followed by reperfusion and vehicle treatment. Group IV HCHF+MCAO+vitamin C: rats were subjected to Rt. MCAO followed by reperfusion and treated with vitamin C at a dose of 250 mg/kg BW. Groups V-VII HCHF+MCAO+OA extracts (OA1, OA2, and OA3): all animals in these groups were subjected to Rt. MCAO followed by reperfusion and treated with OA extracts at doses of 0.5, 5, and 50 mg/kg BW, respectively.

**Figure 1:**
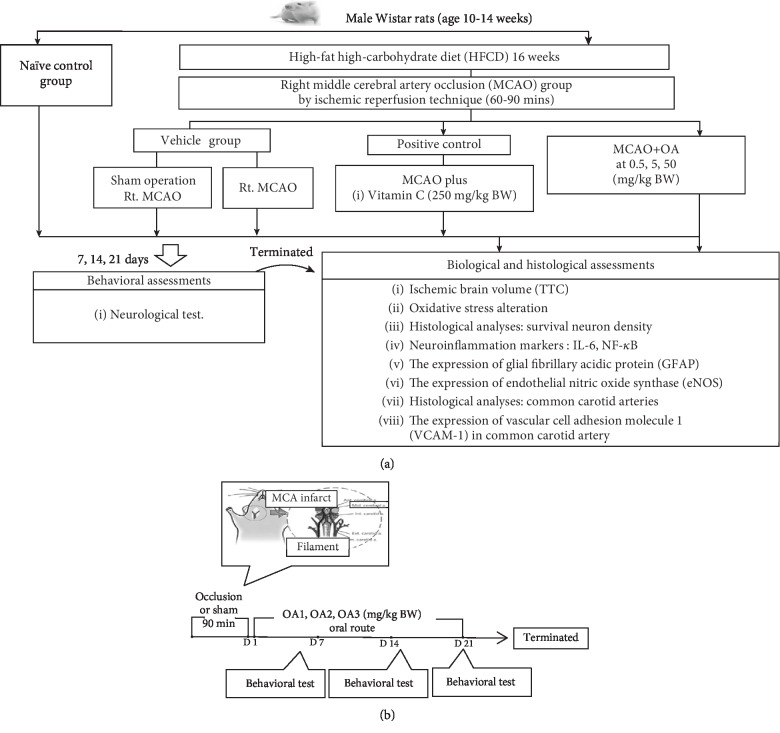
Schematic diagram showing all experimental procedures. (a) Experimental protocol of OA extract treatment and the determination of various parameters. (b) Right MCAO induction and schedule for OA extract treatment. IL-6: interleukin-6; NF-*κ*B: nuclear factor-kappaB; HCHF: high-carbohydrate high-fat diet; MCAO: right middle cerebral artery occlusion; OA1, OA2, and OA3: the combined extract of *O. sativa* and *A. graveolens* at doses of 0.5, 5, and 50 mg/kg BW, respectively.

All animals in groups II-VII were fed with high-carbohydrate high-fat diet comprised of 35.83% of fat, 35.54% of carbohydrate, and 28.6 3% of protein for 16 consecutive weeks in order to induce metabolic syndrome condition. The metabolic syndrome rats (MetS rats) which showed the body weight change more than 40%, fasting plasma glucose (FPG) more than 100 mg/dL, the systolic or diastolic blood pressure higher than 130 and 85 mmHg, respectively, and the atherogenic index higher than the control group were selected for inducing ischemic and reperfusion injury [12]. After the operation, rats were administered the assigned substances once daily for 21 consecutive days via oral route. The neurological score was evaluated every 7 days throughout the study period. At the end of the study, brain infarction volume, the density of survival neuro, and the density of glial fibrillary acidic protein- (GFAP-) positive cells were evaluated. In addition, the oxidative stress status, the nuclear factor-kappa B (NF-*κ*B), the expression of proinflammatory cytokine (IL-6), the endothelial nitric oxide synthase (eNOS) in the cerebral cortex, the expression of vascular cell adhesion molecule 1 (VCAM-1) in the common carotid artery, and the structural changes in the common carotid artery were also determined. The schematic diagram showing the study protocol is shown in Figure 1.

### 2.7. Focal Cerebral Ischemia/Reperfusion Induction

Rats were subjected to an anesthetization by administering pentobarbital sodium at doses of 50 mg/kg BW (Tianjin Kemiou Chemical Reagent Co., Ltd., Tianjin, China) via intraperitoneal route. Following this step, a silicone-coated 4-0 monofilament nylon (USS DG; Tyco Healthcare Group Lp, CT, USA) was gently inserted from the lumen of the right common carotid artery (CCA) and then passed through the internal carotid artery (ICA) approximately 17-18 mm from the bifurcation in order to occlude the right middle cerebral artery. After 90 minutes of intraluminal occlusion, the nylon monofilament was withdrawn to allow the reperfusion process. The identical operation except the intraluminal occlusion was performed in the sham operation group. After operation, rats were cared until they recovered from anesthesia and they were returned to the cage [12].

### 2.8. Neurological Assessment

Neurological score assessment was graded at 24 hours after the induction of reperfusion injury by using the modified neurological severity scores (mNSS), one of the most common methods used in the animal study of stroke. The 18 scale mNSS was performed based on the assessment of neurological functions including motor (muscle status and abnormal movement), sensory (visual, tactile, and proprioceptive), reflex, and balance tests. According to this assessment, the higher score indicated the more severity of neurological deficit [19].

### 2.9. Evaluation of Brain Infarct Volume

After the neurological score assessment, rat brains were removed and prepared as coronal sections at 2 mm thick. The brains were immediately incubated in 2% 2,3,5-triphenyltetrazolium chloride (TTC) solution (Sigma-Aldrich, St. Louis, MO, USA) in the dark at room temperature for 15 minutes and turned over every 5 min. Then, all brain slices were captured using a digital camera (Sony HDR-SR11 Handycam Camcorder; Sony Co. Ltd., Japan), and the infarct size was calculated with the computerized image analysis system [20].

### 2.10. Histological Study

#### 2.10.1. Nissl Staining

The brains of the experimental animals were transcardially perfused with fixative solution containing 4% paraformaldehyde in 0.1 M phosphate buffer pH 7.3 and postfixed in the same fixative overnight at 4°C. Then, they were immersed in a cryoprotectant containing 30% sucrose (Merck, Germany) solution at 4°C for 72 h. Serial sections of the frozen tissues were cut at 20 *μ*m thick using cryostat (Thermo Scientific™ HM525 Cryostat). All sections were placed on slides coated with 0.3% gelatin containing 0.05% aluminium potassium sulfate (Sigma-Aldrich, USA). Following this step, the sections were stained with 0.2% cresyl violet solution (Sigma-Aldrich, USA) for 8 minutes, rinsed with distilled water, and dehydrated with 70%, 95%, and 100% alcohols, respectively (RCI LabScan, Thailand). Then, the sections were immersed in xylene 2 times for 5 minutes each and mounted with DPX mountant (Merck, Germany). The determination of neuron density was performed based on the stereotaxic coordinates from the rat brain atlas as the following: anteroposterior 2.5-4.5 mm and mediolateral 0.2-1.0 mm [21] under the Olympus light microscope model BH-2 (Japan) at 40x magnification. Results were expressed as density of neurons per 255 *μ*m^2^.

#### 2.10.2. Immunohistochemistry

Brain tissue sections for immunohistochemistry evaluation were prepared as mentioned earlier in Section 2.10.1. The prepared sections were immersed in 0.01 M sodium citrate buffer (pH 6.0) and heated using the microwave oven for 10 minutes. After letting cool at room temperature, all sections were washed for 5 minutes with phosphate buffer saline (PBS) (3 times) and incubated in 0.3% hydrogen peroxide for 20 minutes at room temperature. Then, sections were washed for 5 minutes with PBS 3 times and incubated in the solution consisting of 0.3% Triton X-100 (Fluka Chemika, Buchs, Switzerland), 1% (*w*/*v*) bovine serum album (BSA), and 10% normal goat serum for 20 minutes at room temperature. After the incubation process, the sections were washed with PBS (3 times for 5 minutes each) and incubated with primary anti-GFAP (Abcam, Cambridge, MA, USA) at a dilution of 1 : 500 (diluted in the solution containing 0.01 M PBS with 1% Triton X-100 and 10% normal serum) at 4°C overnight. Following this process, sections were washed and incubated with a REAL™ EnVision™ Detection System, peroxidase/DAB+ rabbit/mouse, (Dako, Glostrup, Denmark) for 30 minutes at room temperature. Then, sections were washed with PBS and incubated with 3,3′-diaminobenzidine tetrahydrochloride (DAB) (Sigma-Aldrich, USA) for 5 minutes. Negative control sections were prepared using the identical process without an exposure to primary antibody. Positive staining was shown as a brown color. All sections were mounted on slides coated with gelatin and counterstained with cresyl violet. Then, they were dehydrated with graded alcohols, cleared with xylene, and mounted with DPX mountant. Numbers of positive cells in cerebral cortex were counted by a trained technician who was blind to this experimental design. Results were shown as mean ± SEM.

#### 2.10.3. Hematoxylin and Eosin (H&E) Staining Process

To determine the structural changes of common carotid artery, hematoxylin and eosin (H&E) staining assay was performed. Tissues were fixed in 10% formalin solution and embedded in paraffin. Then, six serial sections (5 *μ*m thick) were prepared and stained with hematoxylin and eosin (H&E). To investigate the detailed histomorphometric changes, the mean diameters of the vessels, tunica intima, and tunica media (*μ*m/vessel) were evaluated [22, 23] by the ImageJ (version 1.52p) program.

### 2.11. Brain Homogenate Preparation

The cerebral cortex used in this study was homogenized in 50 volumes of 0.1 M phosphate buffer saline. The homogenate brains were centrifuged at 3000 g at 4°C for 15 min. The supernatant was kept, and the protein concentration was also measured by using a Thermo Scientific NanoDrop 2000c spectrophotometer (Thermo Fisher Scientific, Wilmington, Delaware, USA).

### 2.12. Biochemical Assessments

#### 2.12.1. Oxidative Stress Marker Assessment

The assessment of thiobarbituric acid reactive substances (TBARSs) was performed in order to measure the level of malondialdehyde (MDA) level, a lipid peroxidation product, in the cerebral cortex [24, 25]. Briefly, the mixture containing 50 *μ*L of 8.1% sodium dodecyl sulfate (SDS) (Sigma-Aldrich, USA), 375 *μ*L of 20% acetic acid (Sigma-Aldrich, USA), 375 *μ*L of 0.8% of thiobarbituric acid (TBA) (Sigma-Aldrich, USA), and 150 *μ*L of distilled water (DW) was mixed with 50 *μ*L of brain homogenate and heated in 95°C boiling water for an hour. After cooling, 250 *μ*L of DW and 1,250 *μ*L of the mixture of n-butanol:pyridine (15,1 *v*/*v*) (Merck, Germany) were added and mixed together. After the 4000 rpm centrifugation for 10 minutes, the separated butanol layer was collected and determined an absorbance at 532 nm. The standard reference was prepared by using the 1,3,3-tetramethoxy propane at the concentration range of 0-15 *μ*M (Sigma-Aldrich, USA). The level of MDA was expressed as ng/mg protein.

The main scavenger enzyme activities including catalase (CAT), superoxide dismutase (SOD), and glutathione peroxidase (GSH-Px) were also determined. Catalase activity was measured via the decrease in the absorbance of H_2_O_2_ [26]. Briefly, 10 *μ*L of enzyme tissue homogenate was mixed with 50 *μ*L of 30 mM of H_2_O_2_ (in 50 mM phosphate buffer, pH 7.0) (BDH Chemicals Ltd., UK), 150 *μ*L of 5 mM KMnO_4_ (Sigma-Aldrich, USA), and 25 *μ*L of H_2_SO_4_ (Sigma-Aldrich, USA). The solution was measured with microplate reading at 490 nm. CAT enzyme (Sigma-Aldrich, USA) at various concentrations ranging from 1 to 100 units/mL was used as a standard reference. Results were expressed in units of catalase per mg protein.

Superoxide dismutase (SOD) activity was determined based on the inhibition rate of cytochrome C reduction by the superoxide radical [27]. Briefly, the cocktail solution contains 57 mM phosphate buffer solution (KH_2_PO_4_) (Sigma-Aldrich, USA), 0.1 mM ethylenediaminetetraacetic acid (EDTA) (Sigma-Aldrich, USA), 10 mM cytochrome C solution (Sigma-Aldrich, USA), and 50 *μ*M of xanthine solution. 200 *μ*L of cocktail solution and 20 *μ*L of 0.5-unit xanthine oxidase were mixed with 20 *μ*L of samples or superoxide dismutase enzyme (Sigma-Aldrich, USA) standard at various concentrations ranging from 1 to 25 units/mL. The absorbance was measured using a microplate reader at 415 nm. Results were presented in units of SOD activity per mg protein.

Glutathione peroxidase (GSH-Px) activity was also assessed [28]. Briefly, the solution containing 10 *μ*L of 1 mM dithiothreitol (DTT) (Sigma-Aldrich, USA) in 6.67 mM potassium phosphate buffer (pH 7), 100 *μ*L of 1 mM sodium azide (Sigma-Aldrich, USA) in 6.67 mM potassium phosphate buffer (pH 7), 10 *μ*L of 50 mM glutathione (Sigma-Aldrich, USA) solution, and 100 *μ*L of 30% hydrogen peroxide (BDH Chemicals Ltd., UK) was mixed with 20 *μ*L of samples. After the exposure to a 5-minute incubation at room temperature, 10 *μ*L of 10 mM DTNB (5,5-dithiobis-2-nitrobenzoic acid) (Sigma-Aldrich, USA) was added. An absorbance was measured using a microplate reader at 412 nm. GSH-Px enzyme (Sigma-Aldrich, USA) at various concentrations ranging from 1 to 50 units/mL was used as a standard reference. GSH-Px activity was expressed as units per mg protein.

### 2.13. Western Blotting Analysis

Samples was homogenized in the solution containing mammalian protein extraction reagent (M-PER) (Pierce Protein Biology Product, Rockford, IL USA) and protease inhibitor cocktail (Sigma-Aldrich, USA) at a ratio of 1 : 10, respectively. After the centrifugation at 12,000 g for 10 minutes at 4°C, the supernatant was harvested. The protein concentration was also determined by using a Thermo Scientific NanoDrop 2000c spectrophotometer (Thermo Fisher Scientific, Wilmington, Delaware, USA). An aliquot of 80 *μ*g of sample lysate was added to Tris-Glycine SDS-PAGE loading buffer (Bio-Rad, USA) with an appropriate concentration and heated at 95°C for 10 minutes. Then, 20 *μ*L of sample protein was loaded onto SDS-polyacrylamide gel and separated by sodium dodecyl sulfate-polyacrylamide gel electrophoresis (SDS-PAGE). In addition, biotinylated broad-range molecular weight markers (Bio-Rad) were also loaded onto the gels. After electrophoresis, samples were transferred to a nitrocellulose membrane, washed with 0.05% TBS-T, and incubated in blocking buffer containing 1% Tween-20 (T-PBS) and 6.5% nonfat dry milk at 4°C overnight. Then, membranes were incubated overnight at 4°C with polyclonal rabbit IL-6 (Cell Signaling Technology, USA; dilution 1 : 1000), anti-NF-*κ*B p65 (Cell Signaling Technology, USA; dilution 1 : 500), anti-eNOS primary antibodies (Cell Signaling Technology, USA; dilution 1 : 1000), and anti-VCAM-1 antibody (Abcam, USA; dilution 1 : 1000). After washing with T-PBS for 30 minutes, they were incubated with anti-rabbit IgG, HRP-linked antibody (Cell Signaling Technology, USA; dilution 1 : 2000) for 1 hour at room temperature. The bands were visualized and quantitated by using the ECL detection systems (GE Healthcare) and the LAS-4000 luminescent image analyzer (GE Healthcare). Band intensities were determined using ImageQuant TL version 7.0 image analysis software (GE Healthcare). The expression was normalized using *β*-actin (Cell Signaling Technology, USA; dilution 1 : 2000). Data were expressed as a relative density to the control normal group.

### 2.14. Statistical Analysis

All data are presented as mean ± SEM. The significant differences among various groups were compared using one-way analysis of variance (ANOVA) followed by the Tukey post hoc test. *p* values of <0.05 were considered to be statistical significant. All statistical data were analyzed using SPSS software version 21.0 (IBM Corp. Released 2012. IBM SPSS Statistics for Windows).

## 3. Results

### 3.1. Changes of Neurological Score and Brain Infarction

**Figure 2:**
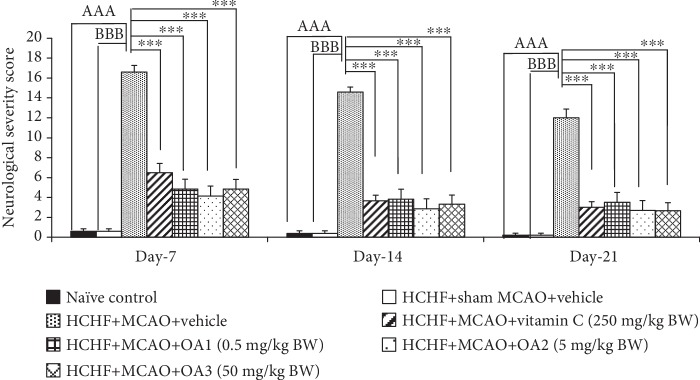
Effect of OA extract on the neurological scores. Data are presented as mean ± SEM. ^AAA^*p* value < 0.001; compared to naïve intact rats, ^BBB^*p* value < 0.001; compared to sham operation which received HCHF diet and vehicle and ^∗∗∗^*p* value < 0.001; compared to MCAO rats which received HCHF and vehicle. HCHF: high-carbohydrate high-fat diet; MCAO: right middle cerebral artery occlusion; OA1, OA2, and OA3: the combined extract of *O. sativa* and *A. graveolens* at doses of 0.5, 5, and 50 mg/kg BW, respectively.

The effect of the combined extract of *O. sativa* and *A. graveolens* on the neurological score is shown in Figure 2. HCHF diet which subjected to sham MCAO failed to produce the significant change on the neurological score throughout a 21-day study period. This suggested that MetS alone did not produce any change on the neurological score. MetS rats which subjected to MCAO and received vehicle showed an increase in the neurological score throughout the study period (*p* value < 0.001 all; compared to the HCHF+sham operation group). Vitamin C significantly decreased the neurological score of MetS rats with MCAO at 7, 14, and 21 days after treatment (*p* value < 0.05, 0.05, and 0.001, respectively; compared to HCHF+MCAO+vehicle). In addition, the neurological scores throughout the study period of MetS rats with MCAO were also improved by the combined extract of *O. sativa* and *A. graveolens* at doses of 0.5, 5, and 50 mg/kg BW (*p* value < 0.001 all; compared to HCHF+MCAO+vehicle).

**Figure 3:**
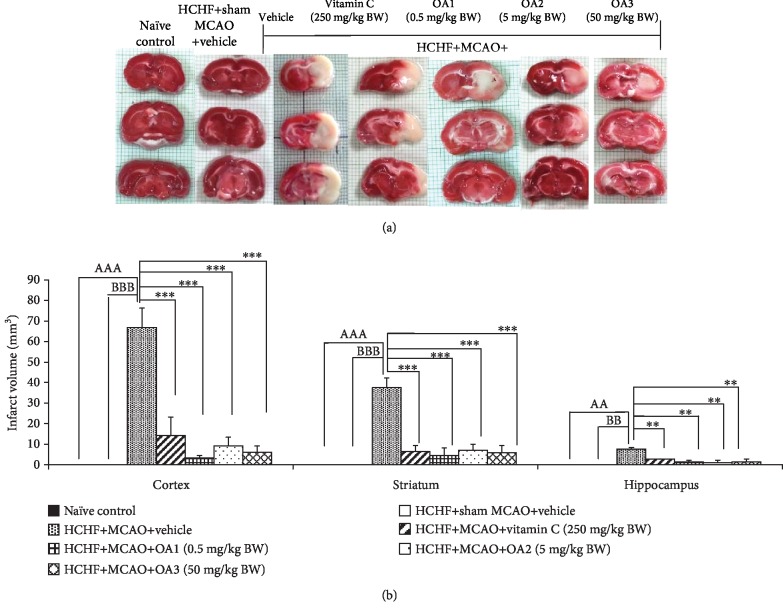
Effect of OA extract on brain infarct volume in the cortex, striatum, and hippocampus. Data are presented as mean ± SEM (*n* = 6/group). ^AA,AAA^*p* value < 0.01 and 0.001; compared to naïve intact rats, ^BB,BBB^*p* value < 0.01 and 0.001; compared to sham operation which received HCHF diet and vehicle and ^∗∗,∗∗∗^*p* value < 0.01 and 0.001; compared to MCAO rats which received HCHF and vehicle. HCHF: high-carbohydrate high-fat diet; MCAO: right middle cerebral artery occlusion; OA1, OA2, and OA3: the combined extract of *O. sativa* and *A. graveolens* at doses of 0.5, 5, and 50 mg/kg BW, respectively.

Based on the correlation between the neurological score and the brain infarcted volume after transient and permanent focal cerebral ischemia in the rat [29], we also determined the changes of brain infarction volume and data are shown in Figure 3. It was found that no infarction volume were observed in the brain of the naïve intact and HCHF diet+sham operation groups as shown in Figure 3(a). However, MCAO induced the infarction area in the brain of MetS rats induced by HCHF diet. Vitamin C and all doses of the combined extract of *O. sativa* and *A. graveolens* significantly decreased the brain infarction area in the cortex, striatum, and hippocampus (*p* value < 0.001 all; compared to HCHF+MCAO+vehicle).

### 3.2. Brain Histological Changes

**Figure 4:**
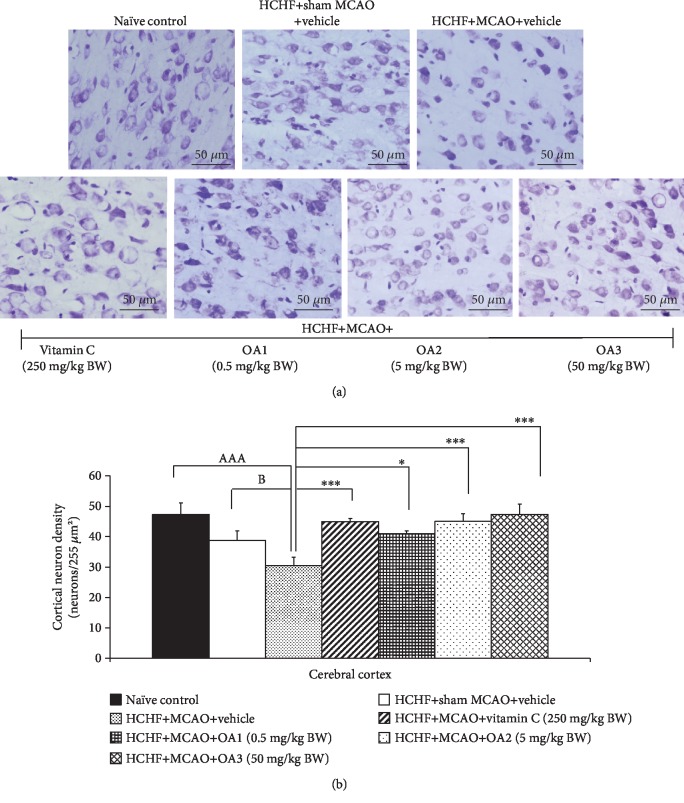
Effect of OA extract on neuron density in the cerebral cortex. (a) Light microscope of coronal sections in the cerebral cortex was stained with cresyl violet at 40x magnification. (b) Density of survival neurons in the cerebral cortex. Data are presented as mean ± SEM (*n* = 6/group). ^AAA^*p* value < 0.001; compared to naïve intact rats and ^∗∗,∗∗∗^*p* value < 0.01, 0.001, respectively; compared to MCAO rats which received HCHF and vehicle. HCHF: high-carbohydrate high-fat diet; MCAO: right middle cerebral artery occlusion; OA1, OA2, and OA3: the combined extract of *O. sativa* and *A. graveolens* at doses of 0.5, 5, and 50 mg/kg BW, respectively.

Previous study had demonstrated that cortical neuronal loss due to brain ischemia induced by the occlusion of MCAO was associated with the behavioral or mood impairments poststroke and it was set up as a target of stroke treatment [30]. Therefore, the neuron density in the cortical area has been investigated, and results are shown in Figure 4. MetS rats without MCAO showed no significant reduction in the cortical neuron density whereas the MetS rats with MCAO and received vehicle showed the significant reduction in the cortical neuron density (*p* value < 0.001; compared to naïve control). This change was mitigated by vitamin C and all doses of the combined extract of *O. sativa* and *A. graveolens* (*p* value < 0.001, 0.05, 0.001, and 0.001, respectively; compared to HCHF+MCAO+vehicle).

**Figure 5:**
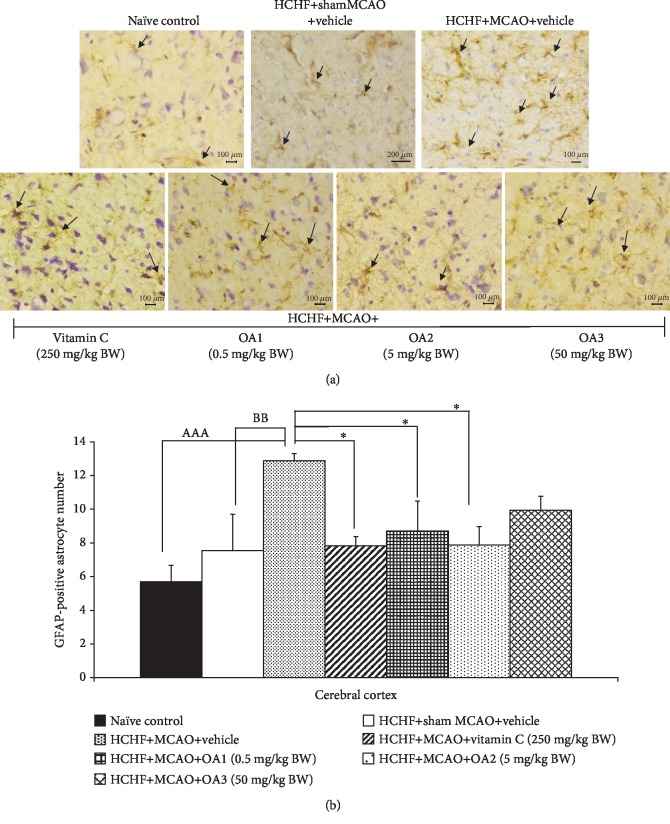
Effect of various doses of OA extract on the density of GFAP-positive cell in the cerebral cortex. (a) Immunostaining for GFAP-positive cell in the cerebral cortex. GFAP-positive cell or astrocytes were stained brown (arrow). Magnification, 40x; scale bar = 100 *μ*m. (b) GFAP-positive cells in the cerebral cortex. Data are presented as mean ± SEM (*n* = 6/group). ^AAA^*p* value < 0.001; compared to naïve intact rats, ^BB^*p* value < 0.01; compared to sham operation which received HCHF diet and vehicle and ^∗^*p* value < 0.05 all; compared to MCAO rats which received HCHF and vehicle. HCHF: high-carbohydrate high-fat diet; MCAO: right middle cerebral artery occlusion; OA1, OA2, and OA3: the combined extract of *O. sativa* and *A. graveolens* at doses of 0.5, 5, and 50 mg/kg BW, respectively. GFAP: glial fibrillary acidic protein.

It has been demonstrated that astrocyte plays an essential role on the recovery of cerebral ischemia due to the physical and chemical inhibitory effect of astrocyte-rich glial scar on the recovery process [31, 32]. Therefore, the effect of the combined extract of *O. sativa* and *A. graveolens* on the changes of astrocyte glial cell was also explored, and results are shown in Figure 5. In this study, GFAP-positive-stained cells were used as an indirect indicator of astrocyte because GFAP is the most important marker of astrocyte [33]. There are no significant change in the density of GFAP-positive-stained cell in the cortical area of MetS rats with sham operation. However, MetS rats with MCAO showed an increase in the density of GFAP-positive-stained cell in the area just mentioned (*p* value < 0.01; compared to HCHF+sham operation). This change was mitigated by vitamin C and the combined extract of *O. sativa* and *A. graveolens* at doses of 0.5 and 5 mg/kg BW (*p* value < 0.05 all; compared to HCHF+MCAO+vehicle). The high dose of the combined extract of *O. sativa* and *A. graveolens* failed to produce the significant change of this parameter.

### 3.3. Brain Biochemical Changes

The effect of the combined extract of *O. sativa* and *A. graveolens* on brain oxidative stress status was also determined due to its important role in brain damage after stroke [34].  Table 1 showed that MetS rats with sham operation failed to produce the significant changes of all oxidative stress markers including MDA, SOD, CAT, and GSH-Px. MCAO significantly decreased the activities of CAT and GSH-Px but increased the MDA level (*p* value < 0.001 all; compared to HCHF+sham operation). Both vitamin C and the medium dose of the combined extract of *O. sativa* and *A. graveolens* increased the activities of SOD, CAT, and GSH-Px enzymes but decreased the MDA level in the cortical area (*p* value < 0.001 all, *p* value < 0.05 all, *p* value < 0.001 all, and *p* value < 0.001 all; compared to HCHF+MCAO). The low dose of the combined extract of *O. sativa* and *A. graveolens* increased the SOD activity but decreased the MDA level (*p* value < 0.001 all; compared to HCHF+MCAO) in the cerebral cortex. However, the high dose of the combined extract of *O. sativa* and *A. graveolens* increased the SOD and CAT activities but decreased the MDA level (*p* value < 0.001, 0.05, and 0.001, respectively; compared to HCHF+MCAO) in the area just mentioned.

**Figure 6:**
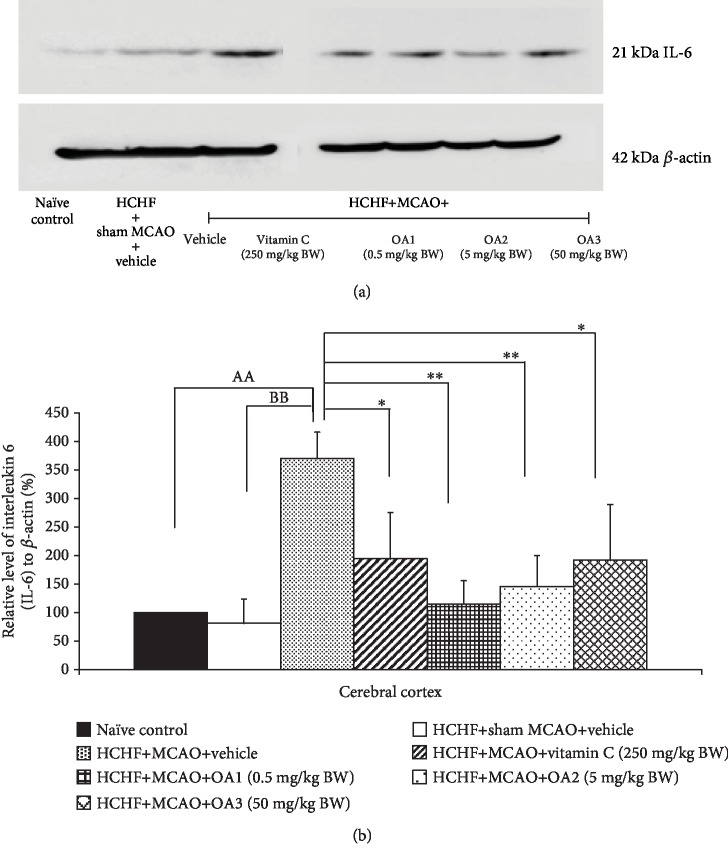
Effect of various doses of OA extract on the expression of IL-6 in the cerebral cortex. (a) Representative western blot showing the levels of IL-6. (b) Relative density of IL-6. Data are presented as mean ± SEM (*n* = 6/group). ^AA^*p* value < 0.01; compared to naïve intact rats, ^BB^*p* value < 0.01; compared to sham operation which received HCHF diet and vehicle and ^∗,∗∗^*p* value < 0.05, 0.01, respectively; compared to MCAO rats which received HCHF and vehicle. HCHF: high-carbohydrate high-fat diet; MCAO: right middle cerebral artery occlusion; OA1, OA2, and OA3: the combined extract of *O. sativa* and *A. graveolens* at doses of 0.5, 5, and 50 mg/kg BW, respectively.

**Figure 7:**
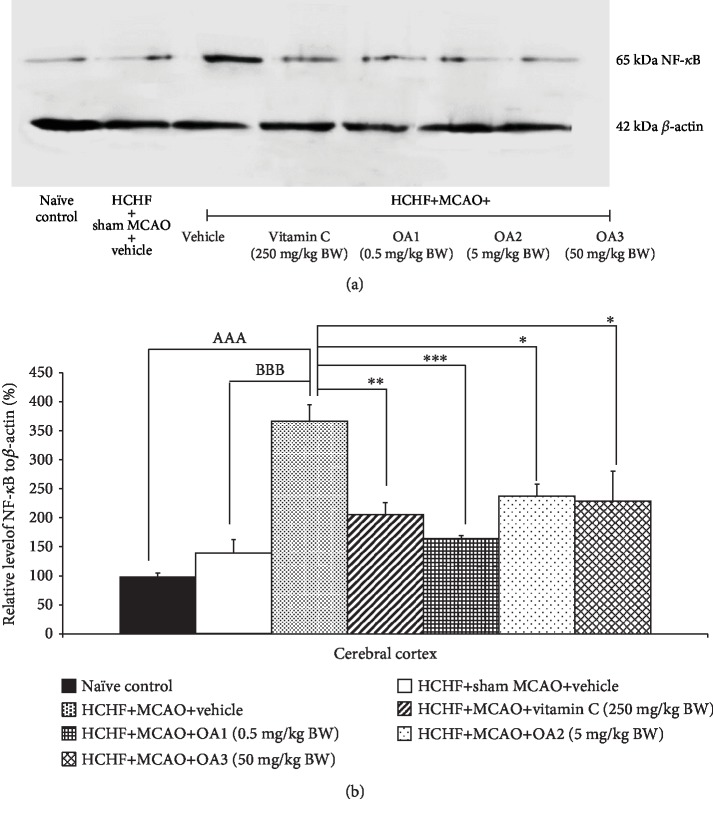
Effect of various doses of OA extract on the expression of NF-*κ*B in the cerebral cortex. (a) Representative western blot showing the levels of NF-*κ*B. (b) Relative density of NF-*κ*B. Data are presented as mean ± SEM (*n* = 6/group). ^AAA^*p* value < 0.01; compared to naïve intact rats, ^BBB^*p* value < 0.01; compared to sham operation which received HCHF diet and vehicle and ^∗,∗∗,∗∗∗^*p* value < 0.05, 0.01, and 0.001, respectively; compared to MCAO rats which received HCHF and vehicle. HCHF: high-carbohydrate high-fat diet; MCAO: right middle cerebral artery occlusion; OA1, OA2, and OA3: the combined extract of *O. sativa* and *A. graveolens* at doses of 0.5, 5, and 50 mg/kg BW, respectively.

The effect of the combined extract of *O. sativa* and *A. graveolens* on the inflammatory cytokines such as IL-6 and NF-*κ*B was also investigated, and data are shown in Figures 6 and 7. MetS rats failed to show the significant modulation effects on the expressions of both IL-6 and NF-*κ*B in the cortical area. However, the increase in the expression of both parameters in MetS rats was observed in MetS rats with MCAO (*p* value < 0.001 all; compared to HCHF+MCAO). Vitamin C and all doses of the combined extract used in this study significantly decreased the expressions of both IL-6 (*p* value < 0.05, 0.01, 0.01, and 0.05; compared to HCHF+MCAO) and NF-*κ*B (*p* value < 0.01, 0.001, 0.05, and 0.04, respectively; compared to HCHF+MCAO) in a cortical area.

**Figure 8:**
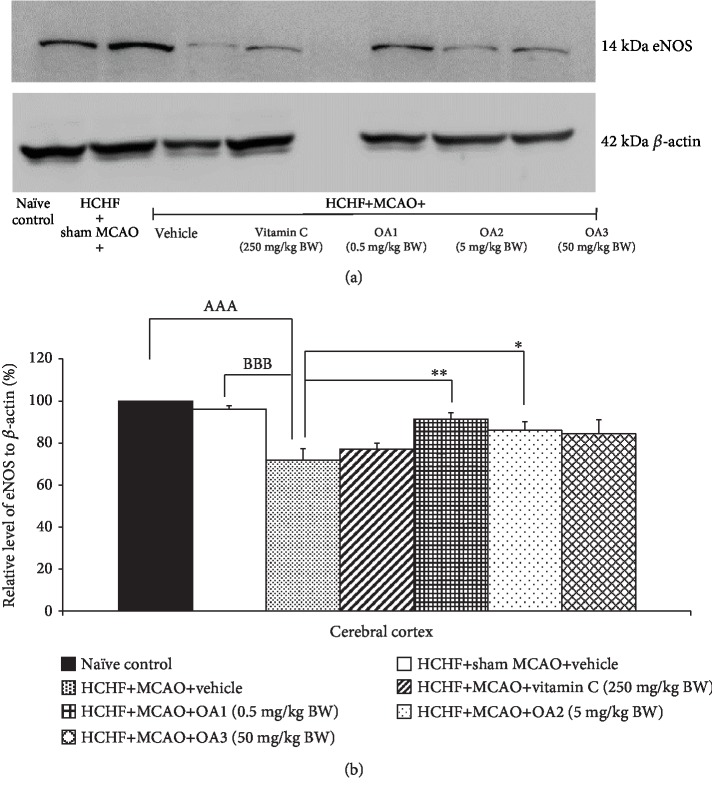
Effect of various doses of OA extract on the expression of endothelial nitric oxide synthase (eNOS) in the cerebral cortex. (a) Representative western blot showing the levels of eNOS. (b) Relative density of eNOS. Data are presented as mean ± SEM (*n* = 6/group). ^AAA^*p* value < 0.001; compared to naïve intact rats, ^BBB^*p* value < 0.001; compared to sham operation which received HCHF diet and vehicle and ^∗,∗∗^*p* value < 0.05, 0.01, respectively; compared to MCAO rats which received HCHF and vehicle. HCHF: high-carbohydrate high-fat diet; MCAO: right middle cerebral artery occlusion; OA1, OA2, and OA3: the combined extract of *O. sativa* and *A. graveolens* at doses of 0.5, 5, and 50 mg/kg BW, respectively.

Figure 8 shows the effect of the combined extract of *O. sativa* and *A. graveolens* on the expression of eNOS on the cortical area. The changes of eNOS in MetS rats with sham operation failed to show the significant effect but the significant reduction of eNOS expression in the cerebral cortex was observed in MetS with MCAO (*p* value < 0.001; compared to HCHF+sham operation). This change was mitigated only in MetS rats with MCAO which received either low or medium dose of the combined extract of *O. sativa* and *A. graveolens* (*p* value < 0.05 and 0.01, respectively; compared to HCHF+MCAO).

### 3.4. Changes of Common Carotid Artery

**Figure 9:**
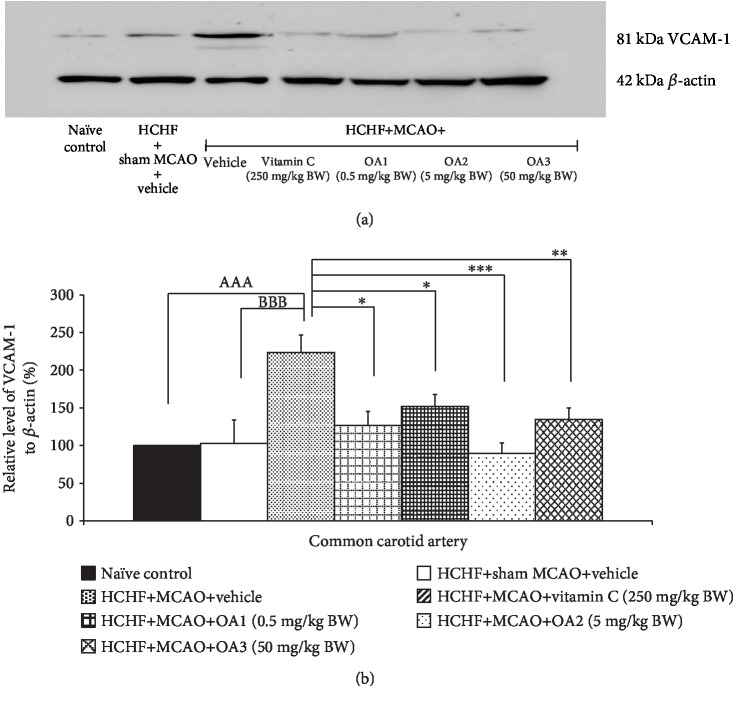
Effect of various doses of OA extract on the expression of vascular cell adhesion molecule 1 (VCAM-1) in common carotid artery. (a) Representative western blot showing the levels of VCAM-1. (b) Relative density of VCAM-1. Data are presented as mean ± SEM (*n* = 6/group). ^AAA^*p* value < 0.001; compared to naïve intact rats, ^BBB^*p* value < 0.001; compared to sham operation which received HCHF diet and vehicle and ^∗,∗∗,∗∗∗^*p* value < 0.05, 0.01, and 0.001, respectively; compared to MCAO rats which received HCHF and vehicle. HCHF: high-carbohydrate high-fat diet; MCAO: right middle cerebral artery occlusion; OA1, OA2, and OA3: the combined extract of *O. sativa* and *A. graveolens* at doses of 0.5, 5, and 50 mg/kg BW, respectively.

**Figure 10:**
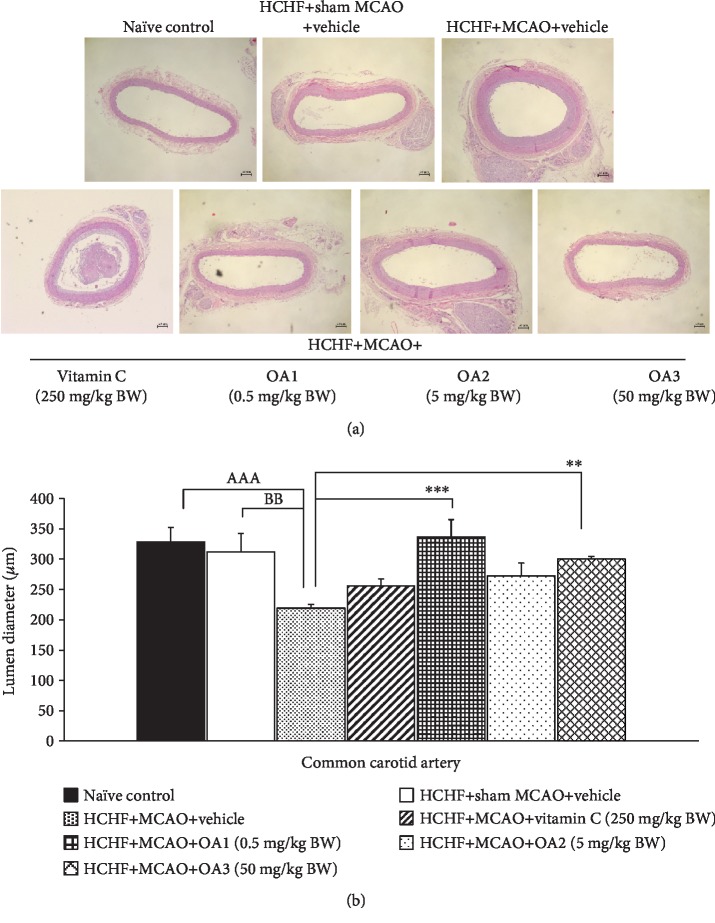
Effect of various doses of OA extract on histopathological appearance of common carotid artery. (a) Light microscope of common carotid artery stained with H&E at 40x magnification. (b) The length of lumen diameter in the common carotid artery. Data are presented as mean ± SEM (*n* = 6/group). ^AAA^*p* value < 0.001; compared to naïve intact rats, ^BB^*p* value < 0.01; compared to sham operation which received HCHF diet and vehicle and ^∗∗,∗∗∗^*p* value < 0.01, 0.001, respectively; compared to MCAO rats which received HCHF and vehicle. HCHF: high-carbohydrate high-fat diet; MCAO: right middle cerebral artery occlusion; OA1, OA2, and OA3: the combined extract of *O. sativa* and *A. graveolens* at doses of 0.5, 5, and 50 mg/kg BW, respectively.

**Figure 11:**
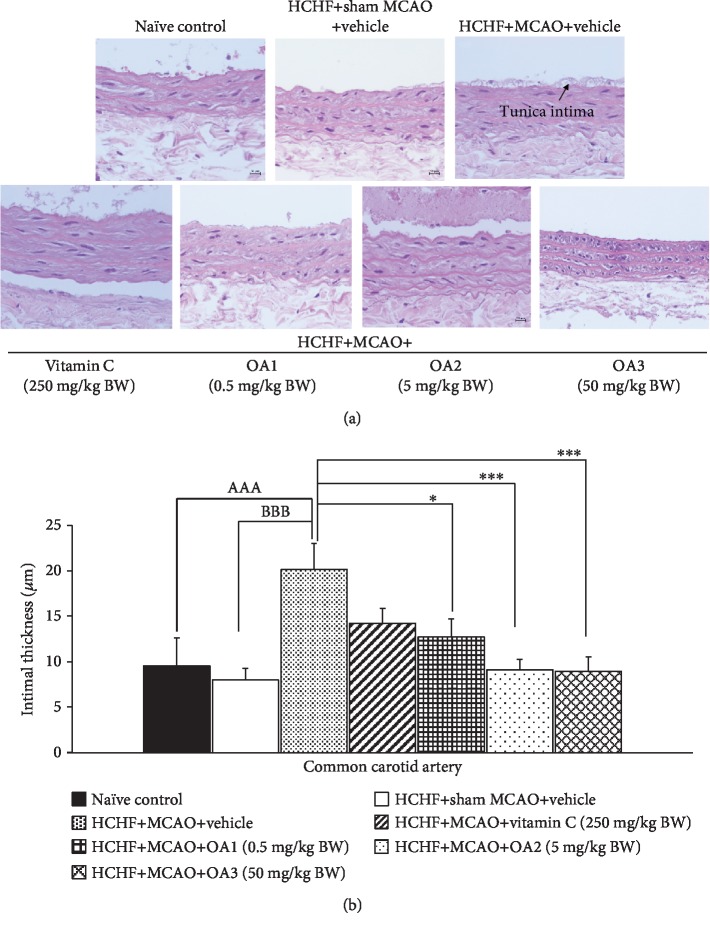
Effect of various doses of OA extract on histopathological appearance of common carotid artery. (a) Light microscope of common carotid artery stained with H&E at 40x magnification. (b) Tunica intima thickness in the common carotid artery. Data are presented as mean ± SEM (*n* = 6/group). ^AAA^*p* value < 0.001; compared to naïve intact rats, ^BBB^*p* value < 0.001; compared to sham operation which received HCHF diet and vehicle and ^∗,∗∗∗^*p* value < 0.05, 0.001, respectively; compared to MCAO rats which received HCHF and vehicle. HCHF: high-carbohydrate high-fat diet; MCAO: right middle cerebral artery occlusion; OA1, OA2, and OA3: the combined extract of *O. sativa* and *A. graveolens* at doses of 0.5, 5, and 50 mg/kg BW, respectively.

**Figure 12:**
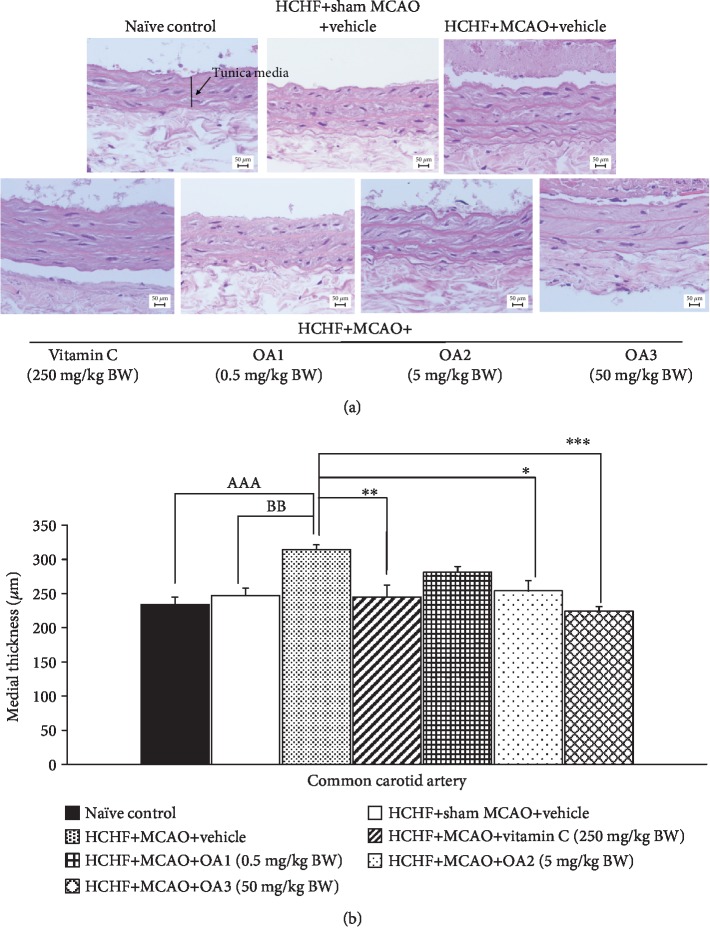
Effect of various doses of OA extract on histopathological appearance of common carotid artery. (a) Light microscope of common carotid artery stained with H&E at 40x magnification. (b) Tunica media thickness in the common carotid artery. Data are presented as mean ± SEM (*n* = 6/group). ^AAA^*p* value < 0.001; compared to naïve intact rats, ^BB^*p* value < 0.01; compared to sham operation which received HCHF diet and vehicle and ^∗,∗∗,∗∗∗^*p* value < 0.05, 0.01, 0.001, respectively; compared to MCAO rats which received HCHF and vehicle. HCHF: high-carbohydrate high-fat diet; MCAO: right middle cerebral artery occlusion; OA1, OA2, and OA3: the combined extract of *O. sativa* and *A. graveolens* at doses of 0.5, 5, and 50 mg/kg BW, respectively.

Although the occlusion of a common carotid artery is a rare case, the common carotid artery is generally associated with occlusion of the distal vessels such as internal carotid artery and middle cerebral artery [35]. Therefore, we also explored the effect of the combined extract of *O. sativa* and *A. graveolens* on the changes of this artery, and results are shown in Figures 9–12. No significant changes in the lumen diameter, the expression of VCAM-1, and the thickness of both tunica intima and tunica media in the common carotid artery of MetS rats were found. MCAO significantly decreased the lumen diameter but increased the expression of VCAM-1 and the thickness of tunica intima and media (*p* value < 0.01, 0.01, 0.001, and 0.01, respectively). Vitamin C treatment could decrease the VCAM-1 proliferation (*p* value < 0.05; compared to HCHF+MCAO) but failed to produce the significant changes in the lumen diameter and the thickness of both tunica intima and media of the common carotid artery. Interestingly, all doses of the combined extract of *O. sativa* and *A. graveolens* produced the significant decrease in VCAM-1 expression (*p* value < 0.05, 0.001, and 0.01, respectively; compared to HCHF+MCAO) and the thickness of tunica intima in the artery (*p* value < 0.05, 0.001, and 0.01, respectively; compared to HCHF+MCAO). The significant reduction in the thickness of the tunica media was observed only in MetS rats which received either the middle or the high dose of the combined extract of *O. sativa* and *A. graveolens* (*p* value < 0.05 and 0.001, respectively; compared to HCHF+MCAO) whereas the significant increase in the lumen diameter was observed only in MetS rats which received the combined extract of *O. sativa* and *A. graveolens* at doses of 0.5 and 50 mg/kg BW (*p* value < 0.001 and 0.01, respectively; compared to HCHF+MCAO).

## 4. Discussion

The current data have demonstrated that the combined extract of *O. sativa* and *A. graveolens* significantly improves neurological deficit in the animal model of cerebral ischemia in MetS condition. The combined extract also decreases the brain infarction, MDA, IL-6, and NF-*κ*B levels but increases the activities of antioxidant enzymes, the expression of eNOS in the frontal cortex. It also increases the lumen diameter but decreases the thickness of both tunica intima and tunica media of the common carotid artery. In addition, the expression of VCAM-1 in the artery just mentioned also decreased.

Our data showed that MetS rats with sham operation failed to show the elevation of MDA whereas the previous study of Obadia and coworkers revealed the increase in brain MDA level. The possible explanation for the discrepancy might be associated with the different type of diet used for MetS induction. Our study used HCHF diet but the previous study used high-fat diet [36]. In addition, the selected cortical area for the investigation might be different because the current study focused on the changes of the frontal cortical area which represented the major area which is affected by the occlusion of MCA, but the previous study did not mention the cortical area that was investigated [37–39]. Aforementioned events contributed the crucial roles on brain infarction and cortical neuronal loss which in turn induced neurological deficit [31, 32, 34, 40]. It was found that cytokines also upregulated adhesion molecule expressions such as ICAM1 and VCAM-1 on the surfaces of cells of the cerebral vasculature which in turn allowed the infiltrate of inflammatory cells in brain parenchyma giving rise to the secondary damage of brain after ischemic reperfusion injury [40]. In addition to the factors mentioned earlier, the changes in blood vessels such as atherosclerosis which in turn played the role on the brain circulation also played the crucial role on the pathophysiology of ischemic stroke [41]. Since the severity of carotid atherosclerosis was a useful indicator of the risk of ischemic stroke [42], the change of adhesion molecule such as VCAM-1 in the common carotid artery was also investigated. VCAM-1 expression was observed in atherosclerotic conditions and early injury [43, 44]. Our data showed that ischemic reperfusion injury also increased the expression of VCAM-1 and the thickness of tunica media and tunica intima of the common carotid artery of MetS rats. Taken all together, MetS rats subjected to ischemic reperfusion injury increased VCAM-1 expression in the artery leading to the infiltrate of inflammatory cells in the blood vessel and the brain parenchyma. Then, the inflammatory cell increased both inflammation and oxidative stress. An elevation of oxidative stress also induced the formation of foam cell at the blood vessel resulting in atherosclerosis and decreased brain blood supply. The formation of foam cell and atheroma gave rise to the increase in intima-media thickness resulting in the reduction of lumen diameter and brain blood supply and brain infarction. In addition, the infiltration of inflammatory cell also produced both oxidative stress and inflammation resulting in infarction.

**Figure 13:**
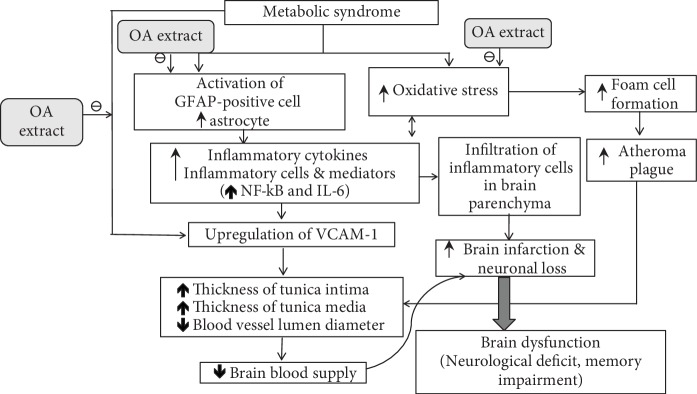
The possible underlying mechanism for the neuroprotective effects of the combined extract of *O. sativa* and *A. graveolens* in an animal model of metabolic syndrome with cerebral ischemic stroke.

In addition, the expansion of ischemic lesions through microcirculatory disturbances after cerebral ischemia also occurred as a result of endothelial dysfunction [45]. Under the normal circumstance, endothelium-derived nitric oxide (eNO), a substance synthesized by endothelial nitric oxide synthase (eNOS), was regarded as an important signal transduction molecule which regulates and maintains brain microcirculation. It was demonstrated that the loss of eNOS gave rise to the reduction in cerebral blood flow resulting in the increase brain infarction area [46, 47]. Therefore, the reduction of eNOS expression in the cortical area observed in this study was also associated with the increased infarction area of the cortex in MetS rats subjected to ischemic reperfusion. Since the downregulation of eNOS was induced by IL-6 [48], we suggested that the suppression of eNOS in the cortical area of MetS rats subjected to MCAO occurred as the result of the elevation of IL-6 in this area. Based on aforementioned information, it has been suggested that substances possessing antioxidant activity such as vitamin C and the combined extract of *O. sativa* and *A. graveolens* can significantly decrease oxidative stress giving rise to the reduction of inflammatory cytokines such as NF-*κ*B and IL-6 which in turn downregulates the expression of VCAM-1 leading to the reduction of intima-media thickness resulting in the increase in lumen diameter of the blood vessel especially the artery and gives rise to the improved brain blood supply and reduced brain infarction and neuronal loss. The reduction in inflammatory cytokines induced by either the antioxidant or the anti-inflammation activities of the combined extract of *O. sativa* and *A. graveolens* [12] also decreases the infiltration of inflammatory cell in the brain parenchyma leading to the improved brain infarction and neuronal loss. In addition, the reduction of IL-6 induced by the combined extract of *O. sativa* and *A. graveolens* also leads to the increase in eNOS expression and the improvement of brain blood supply which in turn decreases brain infarction and neuronal loss. The decreased oxidative stress status induced by the combined extract of *O. sativa* and *A. graveolens* can also decrease the formation of foam cell and atherosclerosis resulting in the improvement of brain infarction and neuronal loss. Moreover, the combined extract of *O. sativa* and *A. graveolens* can also decrease the expression of VCAM-1 directly via the suppression of oxidative stress [49] as shown in Figure 13.

It has been shown that anthocyanin-rich substance can protect against brain damage induced by oxidative stress [12, 50–54]. Therefore, the anthocyanin content in the combined extract of *O. sativa* and *A. graveolens* may contribute the role on the improvement of brain infarction and dysfunction observed in this study. Since it contains numerous ingredients, the lack of a dose-dependent manner can be observed due to masking effect of other ingredients.

## 5. Conclusion

This study has clearly demonstrated that the combined extract of *O. sativa* and *A. graveolens* which is rich in anthocyanins can improve brain infarction and neuronal loss in MetS rats with cerebral ischemia induced by ischemic/reperfusion injury at MCAO. The possible underlying mechanism may occur partly via the reduction in oxidative stress status and inflammation together with the improvement of brain blood supply via the reduction of VCAM-1 and thickness of tunica intima and tunica media. In addition, the increases in eNOS expression in the cortical area and lumen diameter in the artery may also contribute the role. Therefore, the combined extract of *O. sativa* and *A. graveolens* is the potential neuroprotectant against MetS with cerebral ischemia. However, the clinical trial study is essential to confirm the health benefit of this substance.

## Figures and Tables

**Table 1:** The effect of various doses of OA extract on oxidative stress markers in the cerebral cortex.

Treatment group	MDA level (ng/mg·protein)	SOD activity (units/mg·protein)	CAT activity (units/mg·protein)	GSH-Px activity (units/mg·protein)
Naïve control	0.14 ± 0.01	5.30 ± 0.24	5.39 ± 0.61	2.77 ± 0.70
HCHF+sham MCAO+vehicle	0.10 ± 0.02	5.01 ± 0.52	3.88 ± 0.12	1.15 ± 0.19
HCHF+MCAO+vehicle	0.72 ± 0.03^aaa,bbb^	3.11 ± 0.21^aa,b^	2.63 ± 0.19^aaa^	0.51 ± 0.06^aaa^
HCHF+MCAO+vitamin C (250 mg/kg BW)	0.16±0.04^∗∗∗^	6.12±0.83^∗∗∗^	8.36±1.26^∗∗∗^	1.49 ± 0.52^∗^
HCHF+MCAO+OA1 (0.5 mg/kg BW)	0.13±0.02^∗∗∗^	3.55 ± 0.50	7.58±0.74^∗∗∗^	0.93 ± 0.28
HCHF+MCAO+OA2 (5 mg/kg BW)	0.20±0.03^∗∗∗^	4.67±0.94^∗∗^	10.80±0.65^∗∗∗^	1.66 ± 0.18^∗^
HCHF+MCAO+OA3 (50 mg/kg BW)	0.12±0.02^∗∗∗^	3.66 ± 0.69	8.67±2.28^∗∗∗^	1.68 ± 0.09^∗^

Data are presented as mean ± SEM (*n* = 6/group). ^aa,aaa^*p* value < 0.01, 0.001; compared to naïve intact rats, ^b,bbb^*p* value < 0.05, 0.001; compared to sham operation which received HCHF diet and vehicle and ^∗,∗∗,∗∗∗^*p* value < 0.05, 0.01, and 0.001, respectively; compared to MCAO rats which received HCHF and vehicle. HCHF: high-carbohydrate high-fat diet; MCAO: right middle cerebral artery occlusion; OA1, OA2, and OA3: the combined extract of *O. sativa* and *A. graveolens* at doses of 0.5, 5, and 50 mg/kg BW, respectively.

## Data Availability

The data used to support the findings of this study are available from the corresponding author upon request.
